# Low Birth Weight, and Low Ponderal Index Mediates the Association between Preeclampsia, Placenta Previa, and Neonatal Mortality

**Published:** 2020-04

**Authors:** NAWSHERWAN, Abbas KHAN, Nabila BEGUM, Zahoor AHMED, Sumaira MUBARIK, Ijaz Ul HAQ, Ghulam NABI, Naeem ULLAH, Cuifang FAN, Suqing WANG

**Affiliations:** 1. Department of Preventive Medicine, School of Health Sciences, Wuhan University, Wuhan, Hubei, China; 2. Department of Food Science, College of Food Science and Engineering, Jilin University, Changchun, China; 3. Department of Advanced Innovation Center for Food Nutrition and Human Health, Laboratory of Molecular Sensory Science, College of Food and Chemical Engineering, Beijing Technology and Business University, Beijing, China; 4. Department of Food Science, School of Food Science and Engineering, South China University of Technology, Guangzhou, China; 5. Department of Epidemiology and Biostatistics, School of Health Sciences, Wuhan University, Wuhan, Hubei, China; 6. Department of Food Science, College of Food and Pharmaceutical Science, Huaian, Jiangsu, China; 7. Key Laboratory of Animal Physiology, Biochemistry and Molecular Biology of Hebei Province, College of Life Sciences, Hebei Normal University, Shijiazhuang, China; 8. Department of Nutrition and Food Hygiene, School of Public Health, Jilin University, Changchun, China; 9. Department of Obstetrics and Gynecology, Renmin Hospital, Wuhan University, Wuhan, Hubei, China; 10. Hubei Provincial Key Laboratory for Applied Toxicology, Hubei Provincial Academy for Preventive Medicine, Wuhan, Hubei, China

**Keywords:** Low birth weight, Low ponderal index, Preeclampsia, Placenta previa, Neonatal mortality

## Abstract

**Background:**

A retrospective study was conducted to assess the mediation role of low birth weight, and low ponderal index between the preeclampsia, placenta previa, and neonatal mortality in the tertiary hospital of Hubei Province, China.

**Methods:**

A total of 12772 neonatal births were included for data analysis. Birth weight, birth length, and neonatal mortality were recorded after birth. Sobel test based on mediation regression was used to evaluate the effect of mediator variables.

**Results:**

Approximately, 383 (3%) and 409 (3.2%) women experienced preeclampsia and placenta previa respectively. After adjusting for covariates, the indirect effect of preeclampsia on neonatal mortality mediated by low birth weight and low ponderal index was [β 2.59 (95% CI: 0.74 – 4.44)], and [β 3.94 (95% CI: 1.50 – 6.38)] respectively. Moreover, the indirect effect of placenta previa on neonatal mortality mediated by the low birth weight was [β 1.74 (95% CI: 0.16 – 3.31)], and low ponderal index was [β 3.21 (95% CI: 0.95 – 5.48)]. The estimated mediation proportion between the preeclampsia and neonatal mortality accounting for possible mediation by low birth weight and low ponderal index was 44.5% and 34.5% respectively. Furthermore, 47.9% by low birth weight and 33.2% by low ponderal index mediate the association between placenta previa and neonatal mortality.

**Conclusion:**

Low birth weight, and low ponderal index partially mediates the association between preeclampsia, placenta previa and neonatal mortality.

## Introduction

Neonatal mortality is an essential indicator for determining the neonatal health status of a country (1). Globally, approximately, 3 million neonatal deaths have been reported (2). The neonatal time period (0–28 days), has shown the highest mortality in the world (3). The Chinese neonatal mortality contributes about 6.4% among the worldwide neonatal mortality (4). Now adays, due to advancement in technology and social progress, the neonatal death rate is on the declining trend in China (5); however, higher neonatal mortality rate has been reported in the hospitalized cases (6). Among several identified risk factors of neonatal mortality, preeclampsia, and placenta previa have been widely observed to increase the risk of neonatal mortality (7–10).

Preeclampsia is one of the potential causes of maternal and neonatal mortality and morbidity (11). It affects the fetus due to insufficient utero-placental blood flow that leads to adverse neonatal outcomes (12). The prevalence of preeclampsia has been reported as 2–8% of all pregnancies in various countries of the world (13). The etiology of preeclampsia is still elusive. However, maternal obesity, chronic hypertension, kidney disease, diabetes mellitus, and nulliparity are considered the risk factors associated with preeclampsia (14). Placenta previa is one of the abnormal forms of placentation that implants at the lower uterine segment. The incidence of placenta previa in pregnancies is about 0.3% – 0.5% at term gestation (15). It is a significant risk factor for maternal morbidity, mortality, and maternal hemorrhage (16). The abnormal placentation also has an adverse consequence on fetal wellbeing due to premature birth, perinatal mortality, and its undesirable effect on fetal growth (17). Hence, it is the major cause of neonatal morbidity and mortality (18).

Neonates with low birth weight (LBW) are approximately, 20 times more likely to die than heavier neonates (19). LBW remains one of the major causes of neonatal mortality and morbidity (20). LBW can cause early life mortality and development of chronic disease in later life (21). Pregnancy complications such as preeclampsia and placenta previa could be the important risk-factors to the development of LBW (22). Several previous studies have reported the association of preeclampsia, placenta previa with LBW, low ponderal index (LPI), and neonatal mortality but the mediating effect of LBW, LPI between preeclampsia, placenta previa, and neonatal mortality has not been documented in prior published research (7–10, 23–30).

To the best of our knowledge, mediating effect of LBW, LPI between preeclampsia, placenta previa, and neonatal mortality has not examined before in Hubei, China. Therefore, the present study aimed to examine the extent to which low LBW and LPI mediates the association between preeclampsia, placenta previa, and neonatal mortality.

## Materials and Methods

### Study Population

A tertiary hospital-based retrospective study was conducted in the Wuhan University Renmin Hospital, Department of Obstetrics and Gynecology, Hubei, China from January 2011 to March 2017. All the data was collected and documented in obstetrics register by trained nurses during individual medical examination. We excluded 308 with missing data on maternal age, prepregnancy body weight, and neonatal gender (31). A total of 12772 neonatal data were included for data analysis.

### Inclusion and Exclusion Criteria

We included singleton full-term neonates (≥37 weeks) and excluded twins (n=970), preterm (<37 weeks) (n=2450), maternal, and incomplete neonatal record from the data analysis.

### Definitions of Exposure and neonatal outcomes

Pre-eclampsia (PE) defined as the onset of high blood pressure (≥140/90mmHg) and often a significant amount of protein (≥0.3 mg/dL) in urine after 20 weeks of gestation. Placenta previa referred to when the placenta attaches inside the uterus but near or over the cervical opening. LBW is defined as birth weight < 2500g. The ponderal index was determined by weight in gm / (length in cm) ^3^×100. The ponderal index between 2.5 and 3.0 was considered normal, between 2.0 and 2.5 marginal, and a neonate with ponderal index less than 2.0 was considered a LPI. Neonatal mortality is defined as the death of neonate occurs in (0–28 days) after neonatal birth. The neonatal mortality rate was determined by a number of neonatal deaths/number of live births×1000. The Apgar score was determined by evaluating the newborn baby on five simple criteria on a scale from zero to two, then summing up the five values obtained. The Apgar score was recorded at 1 and 5 minutes after birth.

### Ethical Approval

The study was approved by the Ethical Review Board of Renmin Hospital (ID: WDRY2019–K034) Wuhan university in accordance with the Declaration of Helsinki.

### Statistical Analysis

**Fig. 1:**
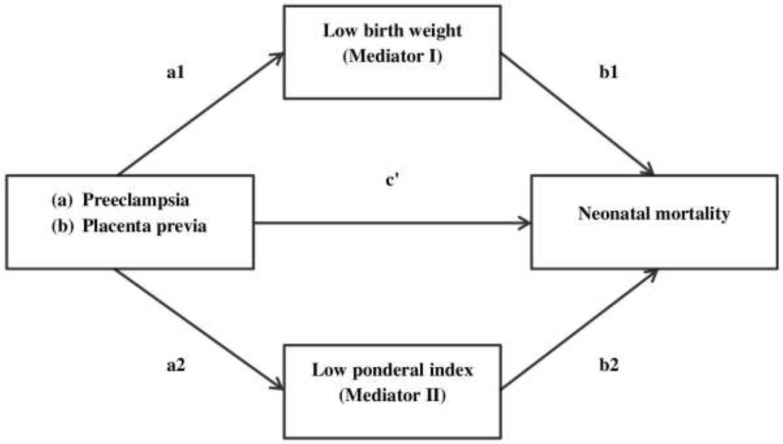
Indirect effect of (a) preeclampsia and (b) placenta previa on neonatal mortality through low birth weight (mediator I) and low ponderal index (mediator II)

We used mediation analysis. In this analysis, the major focus is to determine that how an intermediated variable (mediator/M) mediates the effect of predictor variable (PV) on an outcome variable (OV) (32). Hence, the M lies on the causal pathway between the PV and the OV as shown in Fig 1.

According to the Baron and Kenny statistical mediation approach (33), the total effect (TE) of the PV on the OV is the sum of mediated effect (ME) and direct effect (DE) (34). The ME is the effect of the PV on the OV mediated by the M, whereas the DE is the effect of the PV on the OV keeping the M constant. We used the Sobel test of mediation (35) and regression-based approach by implementing regression mediation models proposed by Imai et al. (36) and MacKinnon (37). The hallmark of this regression-based mediation approach is that confounding variables and covariates can be included in the models.

The ME was estimated by multiplying the regression coefficient of the effect of the PV on the M from Model 2/M2 and Model 3/M3 with the regression coefficient of the effect of the M on the OV from Model 4/M4 (33, 37). One of the best ways of expressing ME is by determining the “mediation proportion (MP),” which is the proportion of the TE explained by a particular M (34, 36, 37). The MP was determined by a theoretical model as $1-\frac{c^{\prime }}{c}$ proposed by Baron and Kenny [33]. Whereas (*c*) represents the TE (sum of DE and ME) of PV on OV and *c*′ represents the ME of PV on OV with M included as a covariate, which is obtained from (M1, M2, M3, M4). The 95% confidence intervals for the ME were calculated by the bootstrap approach (38, 39). *P* <0.05 was taken statistically significant. The analysis was performed using SPSS (Chicago, IL, USA) and R software.

## Results

### Maternal and Neonatal Characteristics

Our analysis consisted of a total 12772 women. Around, 383 (3%) and 409 (3.2%) women experienced preeclampsia and placenta previa respectively. Neonates born with LBW were 358 (2.8%) while, 294 (2.3%) were born with a LPI. Almost, 12005 (94%) neonates had normal (>7), 510 (4%) intermediate (4–6), and 257 (2%) low (0–3) APGAR score. The neonatal mortality rate was found at 8.7 per 1000 live birth (Table 1).

**Table 1:** Maternal and neonatal characteristics of study population (n= 12772)

***Variables***		***(Mean±SD), Frequency (%)***
Maternal age (yr)		29.6±4.3
Neonatal sex	Male	6884 (53.9)
	Female	5888 (46.1)
APGAR score	>7	12005 (94)
	4–6	510 (4)
	0–3	257 (2)
Ponderal index	Normal	9860 (77.2)
	Marginal	2618(20.5)
	Lower	294 (2.3)
Low birth weight		358 (2.8)
Pre-eclampsia		383 (3)
Placenta previa		409 (3.2)
Diabetes		281 (2.2)
GDM		665 (5.2)
Cesarean section		7791 (61)
Multigravidity		6143 (48.1)
Multiparity		3627 (28.4)
Neonatal mortality/ 1000 live birth		8.7/1000

### Mediation Analysis of Neonatal Outcomes

The Sobel test results showed LBW and LPI mediate the association between preeclampsia, placenta previa, and neonatal mortality (Table 2).

**Table 2:** Sobel Test of Mediation of Analysis

***Variable***	***Input***		***Test Statistics***	**P-*value***
a^*^	1.79			
b^*^	1.83			
Sa	0.17	Sobel Test	4.28	0.000
Sb	0.39			
a^**^	1.41			
b^*^	1.83			
Sa	0.22	Sobel Test	3.78	0.000
Sb	0.39			
a^***^	2.13			
b^**^	1.64			
Sa	0.32	Sobel Test	2.76	0.005
Sb	0.54			
a^****^	1.84			
b^**^	1.64			
Sa	0.36	Sobel Test	2.61	0.009
Sb	0.54			

**Note:**

where a^*^, a^**^ = Coefficients of preeclampsia and placenta previa respectively independent variable (IV) and low birth weight as a dependent variable (DV), b^*^ = Coefficient of low birth weight (mediator variable) and neonatal mortality (DV), where a^***^, a^****^ = Coefficients of preeclampsia and placenta previa respectively (IV) and low ponderal index (as a DV), b^**^ = Coefficient of low ponderal index (mediator variable) and neonatal mortality (DV), Sa = standard error of a, Sb = standard error of b

The adjusted odds ratio (aOR), and p-value from the regression models (M1, M2, M3 and M4) are shown in table 3. In M1, preeclampsia [aOR 8.02 (95% CI: 3.20 – 20.12)] and placenta previa [aOR 4.99 (95% CI: 1.87 – 13.33)] had significant association with neonatal mortality (Table 3 (a)). In M2, preeclampsia [aOR 7.28 (95% CI: 4.89 – 10.84)] and placenta previa [aOR 3.79 (95% CI: 2.37 – 6.04)] had a significant association with LBW (Table 3 (b)). Moreover, in M3, preeclampsia [aOR 9.79 (95% CI: 5.13 – 18.69)] and placenta previa [aOR 6.47 (95% CI: 3.08 – 13.60)] had also a significant association with LPI (Table 3 (c)). When preeclampsia [aOR 5.56 (95% CI: 2.12 – 14.58)], placenta previa [aOR 4.38 (95% CI: 1.64 – 11.71)], LBW [aOR 3.72 (95% CI: 1.57 – 8.79)] and LPI [aOR 5.69 (95% CI: 1.65 – 19.51)], were considered as predictors of neonatal mortality (M4), all variables were found to be statistically significant (Table 3 (d)).

**Table 3:** Mediation regression analysis of low birth weight and low ponderal index between preeclampsia, placenta previa and neonatal mortality

***Variables***	***β (Coef)***	***aOR***	**P-*value***
Preeclampsia	2.08	8.02	0.000
Placenta previa	1.60	4.99	0.001
Preeclampsia	1.98	7.28	0.000
Placenta previa	1.33	3.79	0.000
Preeclampsia	2.28	9.79	0.000
Placenta previa	1.86	6.47	0.000
Preeclampsia	1.71	5.56	0.000
Placenta previa	1.47	4.38	0.003
low birth weight	1.31	3.72	0.003
Low ponderal index	1.73	5.69	0.006

**Note:**

NM (Neonatal Mortality), Pre (Preeclampsia), Plac (Placenta previa), LBW (Low Birth Weight), LPI (Low Ponderal Index), ^*^Adjusted for maternal age, prepregnancy body weight, maternal year of birth, diabetes, gestational diabetes mellitus, cesarean section, multiparity, low APGAR score, and neonatal gender

### Mediated Effect and Mediation Proportion

From the mediation regression analysis, we observed ME of preeclampsia on neonatal mortality mediated by LBW and LPI was [β 2.59 (95% CI: 0.74 – 4.44)], and [β 3.94 (95% CI: 1.50 – 6.38)] respectively. Moreover, the ME of placenta previa on neonatal mortality mediated by LBW was [β 1.74 (95% CI: 0.16 – 3.31)], and LPI was [β 3.21 (95% CI: 0.95 – 5.48)]. The estimated MP between preeclampsia and neonatal mortality accounting for possible mediation by LBW and LPI was 44.5% and 34.5% respectively. Furthermore, 47.9% by LBW and 33.2% by LPI mediates the association between placenta previa and neonatal mortality (Table 4).

**Table 4:** Indirect effects /mediated effects (mediated by low birth weight and low ponderal index) of preeclampsia and placenta previa on neonatal mortality

***Outcome***	***Mediated effect/ME***	***% Mediated***
Neonatal mortality [β, 95% CI]	^a*^ 2.59 (0.74 – 4.44)	44.5 %
	^a**^3.94 (1.50 – 6.38)	34.5 %
	^b*^ 1.74 (0.16 – 3.31)	47.9 %
	^b**^3.21 (0.95 – 5.48)	33.2 %

Note:

where a^*^, a^**^= mediated effect of preeclampsia on neonatal mortality mediated by (low birth weight and low ponderal index and) respectively, b^*^, b^**^= mediated effect of placenta previa on neonatal mortality mediated by (low birth weight and low ponderal index) respectively.

## Discussion

To study the association between PV and OV, there are certain conditions in which the PV affects the OV both directly and indirectly, through an intermediate variable (M), which further influences OV. Therefore, considering mediators in statistical analysis enables researchers to fully understand the complex association between variables.

### Preeclampsia and Neonatal Outcomes

In patients with preeclampsia, the utero-placental blood perfusion drops to 50–60 % after 3 to 4 weeks of the complication. A shallow trophoblastic invasion of the decidual arteries can cause pre-eclampsia, and the hypo-uteroplacental flow cause insufficient transport of the nutrients. It is intuitive that hypo-uteroplacental blood flow should induce decreased fetal growth, with an increased risk of LBW and intrauterine growth restriction (40).

From mediation regression model, we found that preeclampsia significantly (*P*< 0.05) increased the risk of LBW and LPI. A significant negative association between the preeclampsia and neonatal LBW is reported (41, 42). Moreover, preeclampsia increased the risk of neonatal LBW, intrauterine growth restriction, and LPI (23, 24). Furthermore, consistent with our results, a large population-based study conducted in Norway reported an increased risk of LBW and the LPI associated with preeclampsia (25).

We also observed that preeclampsia significantly (p< 0.05) increased the risk of neonatal mortality. Among obstetric complications, preeclampsia was the leading cause of neonatal mortality (7). Another hospitalized based study also found that preeclampsia was the primary cause of neonatal mortality (8). These two studies were conducted in a different population with different sample sizes; however, they give a similar picture of a very important contribution of preeclampsia to neonatal mortality. Furthermore, neonates born to preeclampsia mothers had higher neonatal mortality compared to neonates born to mothers without preeclampsia (43). In China, a significantly higher numbers of early neonatal deaths were noted in the preeclampsia group (44).

### Placenta Previa and Neonatal Outcomes

Placenta previa is one of the abnormal forms of placentation that hurts fetal wellbeing due to morbidity and perinatal mortality, in particular, its undesirable effect on fetal growth (17). Some factors might be behind its effect on fetal growth. Firstly, the blood flow to the lower uterine cavity is less compared to the upper uterine cavity which results in hypo-fetoplacental blood perfusion (45). Secondly, on-and-off bleeding from placenta previa may affect fetal oxygenation and fetal growth (9).

We observed that placenta previa significantly increased (*P*<0.05) the risk of LBW and LPI. The association between placenta previa and LBW is mainly due to the influences of preterm birth, and virtually very little due to impaired fetal growth (46). However, our study included only term neonates and observed that placenta previa increased the risk of LBW. It suggests that the association between placenta previa and LBW might be equally due to the influences of premature birth and fetal growth restriction. In different populations, placenta previa was the independent risk factor for LBW (26–29). The findings of these studies are in line with our current results.

We found that placenta previa was a significant (*P*<0.05) risk factor of neonatal mortality. Prior studies have reported contradictory results on the association between placenta previa and neonatal mortality (9, 30). Placenta previa was a significant risk factor for neonatal mortality. However, that study also included cases of thrombophilia, diabetes, and preeclampsia, which may be associated with adverse neonatal outcomes (9). On the other hand, placenta previa was not associated with neonatal mortality. However, in the placenta previa group, lower placental weight was associated with neonatal mortality (30). Furthermore, placenta previa was significantly associated with neonatal mortality (10). In the author’s opinion, neonatal mortality was because of delay in arrival and severe anemia.

### Neonatal Mortality Rate

We found that the neonatal mortality rate was 8.7 /1000 live birth. In 2015, the neonatal mortality rate was 0.9/1000 live births in Japan, 3.6/1000 live births in the US, and 5.5/1000 live births in China. In China, the major causes of neonatal mortality were LBW, preterm birth (28–32 weeks), infection, asphyxia, and neonatal respiratory distress syndrome (47). Moreover, in our findings, the neonatal mortality rate was higher than the previous report (47) maybe because of the monocentric tertiary hospital-based study. In general, high-risk pregnant women delivered more often in the tertiary hospital, which in turn increases the risk of neonatal mortality. We acknowledge that our study had certain limitations.

To eliminate the effect of preterm birth and twins on the LPI, LBW, and neonatal mortality, we confined our analysis to only singleton term birth, which is the potential selection bias in our analysis. The study was conducted in only one tertiary hospital. So, our results cannot be generalized to the whole population.

## Conclusion

LBW and LPI partially mediate the association between preeclampsia, placenta previa, and neonatal mortality. Furthermore, large population-based study is required to confirm our results.

## Ethical considerations

Ethical issues (Including plagiarism, informed consent, misconduct, data fabrication and/or falsification, double publication and/or submission, redundancy, etc.) have been completely observed by the authors.
